# Managing a difficult airway due to supraglottic masses: successful videolaryngoscopic intubation after induction of general anesthesia

**DOI:** 10.1186/s13741-024-00377-9

**Published:** 2024-03-20

**Authors:** Hye-won Jeong, Eun-Jin Song, Eun-A Jang, Joungmin Kim

**Affiliations:** 1 Department of Anesthesiology and Pain Medicine, Chonnam National University Hospital, Gwangju, Korea; 2 Department of Anesthesiology and Pain Medicine, Chonnam National University Hwasun Hospital, Hwasun, Korea; 3 Department of Anesthesiology and Pain Medicine, Chonnam National University School of Dentistry, Gwangju, Korea; 4 Department of Anesthesiology and Pain Medicine, Chonnam National University Medical School, Gwangju, 61469 South Korea

**Keywords:** Difficult airway management, Videolaryngoscopy, Intubation, General anesthesia, Neuromuscular blockade, Supraglottic masses, Multidisciplinary approach

## Abstract

**Background:**

While awake, flexible bronchoscopic intubation has long been considered the gold standard for managing anticipated difficult airways, the videolaryngoscope has emerged as a viable alternative. In addition, the decision to perform awake intubation or to proceed with airway management after induction of general anesthesia should be grounded in a comprehensive assessment of risks and benefits.

**Case presentation:**

A 41-year old female patient was scheduled for excision of bilateral, mobile, and pedunculated masses on both aryepiglottic folds, which covered almost the entire upper part of the glottis. We conducted a comprehensive evaluation of the patient’s signs and symptoms, which included neither stridor nor dyspnea in any position, along with the otolaryngologist’s opinion and the findings from the laryngeal fiberscopic examination. Given the potential challenges and risks associated with awake flexible bronchoscopic intubation for this patient, we decided to proceed with gentle tracheal intubation using a videolaryngoscope under general anesthesia. In case of failed mask ventilation and tracheal intubation, we had preplanned strategies, including awakening the patient or performing an emergent tracheostomy, along with preparations to support these strategies. Ensuring that mask ventilation was maintained with ease, the patient was sequentially administered intravenous propofol, remifentanil, and rocuronium. Under sufficient depth of anesthesia, intubation using a videolaryngoscope was successfully performed without any complications.

**Conclusions:**

Videolaryngoscopic intubation after induction of general anesthesia can be a feasible alternative for managing difficult airways in patients with supraglottic masses. This approachshould be based on a comprehensive preoperative evaluation, adequate preparation, and preplanned strategies to address potential challenges, such as inadequate oxygenation and unsuccessful tracheal intubation.

## Background

Endotracheal intubation in patients with a supraglottic mass presents a significant challenge for anesthesiologists. The presence of a large supraglottic mass can impede mask ventilation and tracheal intubation, leading to potential difficulties in airway management (Choi et al. 2010, Liew et al. 2015, Min Lee and Lim 2019). Therefore, comprehensive preoperative evaluation, preparation, and preplanned strategies are essential for managing anticipated difficult airways (Apfelbaum et al. 2022).

Awake flexible bronchoscopic intubation is regarded as the technique of choice for patients with anticipated difficult airways (Larson and Parks 1988, Sidhu et al. 1993). However, achieving a smooth and successful process of awake bronchoscopic intubation, which includes the application of topical anesthesia and flexible bronchoscopic manipulation, can be particularly challenging in patients with upper airway obstructions (Mason and Fielder 1999). Therefore, the decision to perform awake intubation or manage the airway after induction of general anesthesia should be made on a case-by-case basis by considering the individual risk-benefit ratio (Apfelbaum et al. 2022). Furthermore, recent studies have reported comparable effectiveness of the videolaryngoscope and flexible bronchoscope for awake tracheal intubation; similar outcomes are achieved in terms of glottic view, laryngeal inlet visualization time, intubation success rate on the first attempt, and intubation time (Abdellatif and Ali 2014, Nassar et al. 2016, Rosenstock et al. 2012).

This case report illustrates a successful intubation process using a videolaryngoscope under general anesthesia in a patient with bilateral, mobile, and pedunculated masses on both aryepiglottic folds.

## Case presentation

**Fig. 1:**
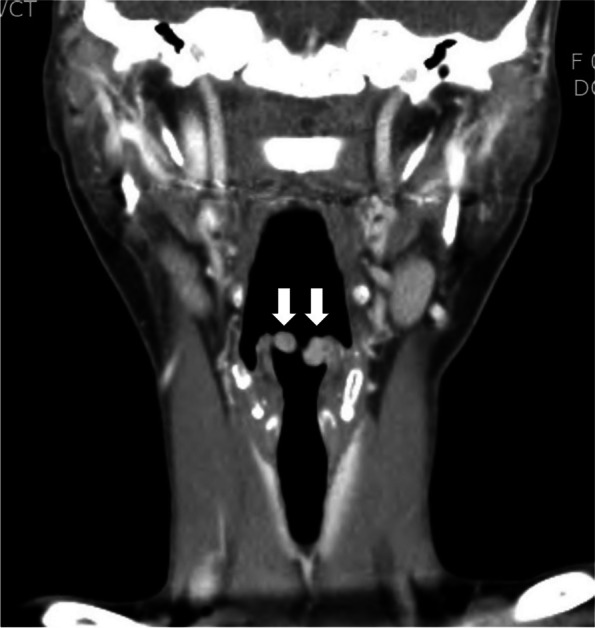
Coronal computed tomography scan of the neck shows bilateral enhancing nodular lesions (arrows) arising from both aryepiglottic folds

**Fig. 2:**
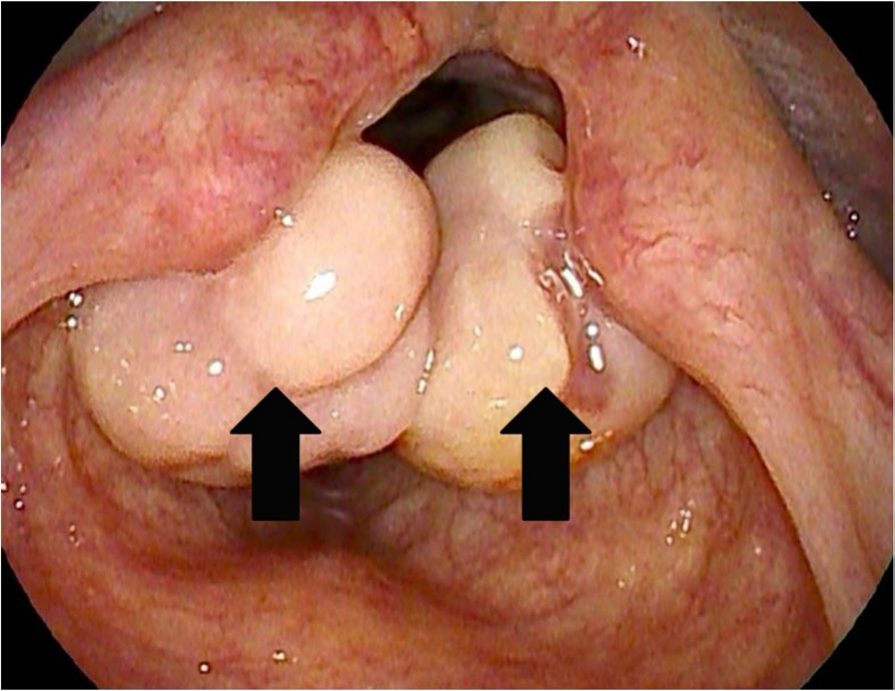
Laryngeal fiberscopic view during preoperative laryngeal examination. Bilateral masses (arrows) arising from both aryepiglottic folds could be observed

This case report was approved by the Institutional Review Board of the Chonnam National University Hospital (IRB no. 20112). A written informed consent for publication was obtained from the patient. A 154-cm, 47-kg, 41-year-old female patient presented to the ear, nose, and throat (ENT) outpatient department with a 2-month history of hoarseness and dysphagia. She had no other underlying disease and could perform daily activities without limitations. On clinical examination, she exhibited hoarseness but did not show any signs of stridor or coughing. She had not experienced shortness of breath in any position and was able to take deep breaths. Laryngeal fiberscopic examination revealed the presence of bilateral, mobile, and pedunculated masses on both aryepiglottic folds. These masses partially covered the upper part of the glottis. Computed tomography of the neck revealed a mass 0.8 cm in diameter originating from the right aryepiglottic fold and a mass 1.0 cm in diameter originating from the left aryepiglottic fold (Fig. 1). She was scheduled to undergo laryngeal microsurgery under general anesthesia 1 month later. However, during the follow-up conducted 2 weeks before surgery, the masses had increased considerably in size, covering almost the entire upper part of the glottis (Fig. 2). Due to the rapid growth rate, the surgery was rescheduled for 2 days later.

As part of our routine preoperative evaluation of patients with upper airway masses or polyps, we discussed this patient’s airway management issues with ENT surgeons. In the meeting, we focused on the potential challenges of mask ventilation and the risk of damaging the masses during endotracheal intubation. The experienced ENT surgeon, who had assessed the patient’s signs and symptoms and performed a direct laryngeal fiberscopy, provided detailed insights into the characteristics of the masses. It was concluded from a comprehensive evaluation that the surgeon’s primary concern was the potential dislodgement of the narrow-stalked mass during airway management, rather than maintaining airway patency. If the very narrow stalks of the masses on either side of the aryepiglottic folds were cut off, parts or all of the masses could move past the vocal cords, causing distal airway obstruction. This risk might be heightened if either the topical anesthesia for awake intubation or the process of endotracheal intubation using a flexible bronchoscope was not performed smoothly. Integrating the surgeon’s opinion with our preoperative assessment, we prioritized preventing dislodgement of the masses during the entire anesthesia process while ensuring airway patency. Given that the patient had never exhibited stridor or dyspnea in any position and based on the findings from the laryngeal fiberscoptic examination, the initial decision was to proceed with a gentle tracheal intubation using a KoMAC videolaryngoscope (KoMAC Co., Ltd., Seoul, Republic of Korea) after induction of general anesthesia with neuromuscular blockade.

A preoperative airway assessment revealed a Mallampati score of 1 with normal mouth opening and neck movements. The patient was informed about the potential risk of undergoing an emergent tracheostomy if mask ventilation or tracheal intubation was not possible during the induction of general anesthesia. The patient understood and consented to undergo any necessary procedures.

The patient was taken to the operating room without premedication, and standard monitoring was initiated. Prior to the induction of general anesthesia, we prepared for potential challenges such as insufficient oxygenation or failed tracheal intubation during the process; we had 16 mg of sugammadex ready for the rapid reversal of rocuronium. The ENT surgeon marked the tracheostomy site and remained on standby with a tracheostomy set, ready for immediate use if necessary. In addition, to address the possibility of masses dislodged into the bronchus and causing distal airway obstruction, the ENT surgeon had a rigid bronchoscope prepared for the removal of the masses.

Preoxygenation was conducted using a closed face mask with 100% oxygen delivered at a rate of 8 L/min for more than 5 min. After confirming that an end-tidal oxygen concentration of 90% had been achieved, induction of general anesthesia was started. The difficulty in achieving manual mask ventilation was assessed using the Han scale for mask ventilation (Han et al. 2004). The administration of medications was sequential, ensuring that lung ventilation was maintained with ease. An initial dose of 50 mg of 1% propofol was administered, and mask ventilation was assessed (Han grade 1: ventilation by mask without adjuncts). Additionally, a 50-mg dose of 1% propofol was given, resulting in patient unresponsiveness, and ventilation was reassessed (Han grade 1). Subsequently, 40 mg of rocuronium was administered, and successful mask ventilation was confirmed (Han grade 1). The initial rate of remifentanil infusion was 0.1 μg/kg/min, and it was gradually increased to 0.3 μg/kg/min.

**Fig. 3:**
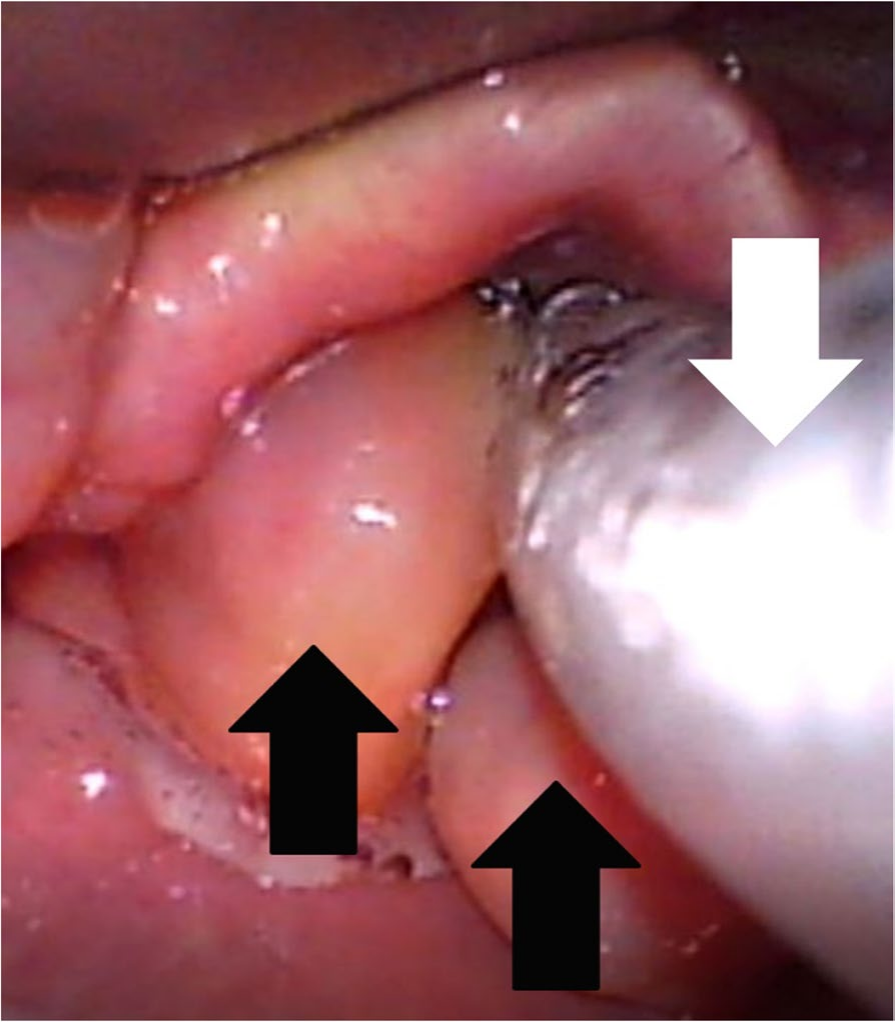
Videolaryngoscpic view following a successful tracheal intubation. An armored endotracheal tube (white arrow) was inserted between the bilateral aryepiglottic fold masses (black arrows) into the glottal opening

When anesthesia was considered to be deep enough to suppress upper airway reflexes, such as coughing and laryngospasm, the KoMAC videolaryngoscope blade was gently inserted into the patient’s mouth. The tip of the blade was carefully positioned in the vallecula, and the glottal opening was visible below the bilateral masses. The structures around the vocal cords, including the masses, were clearly visible to both the attending anesthesiologist and assistant on the videolaryngoscope monitor. A prompt decision was made to proceed with tracheal intubation instead of waking the patient or performing an emergency tracheostomy. It was also decided that tracheal intubation attempts should be limited to a maximum of 1–2 times to minimize the potential for bleeding or damage of the masses. We confirmed to use a 5.5-mm armored endotracheal tube (ETT), which had been preloaded with a stylet. By carefully advancing the tip of the ETT between the bilateral masses towards the glottal opening under continuous video control, successful tracheal intubation was achieved (Fig. 3). We visually confirmed the successful passage of the ETT without significant damage or bleeding of the masses during the procedure. An ETT with a smaller diameter than usual was chosen to reduce the risk of disrupting the masses and to facilitate the smooth passage of the tube into the glottal opening. Anesthesia and airway management took about 10 min; throughout this time, oxygenation and hemodynamics remained stable. Anesthesia was maintained with desflurane and remifentanil.

Surgery took place over the next 50 min. The ENT surgeon performed a biopsy and excision of the bilateral aryepiglottic fold masses using microscissors. Throughout the operation, the patient’s vital signs remained stable, and no notable findings were observed. At the end of the surgery, videolaryngoscopy was used to verify the absence of edema or bleeding at the surgical site. After confirming the patient’s recovery of consciousness and spontaneous breathing, extubation was performed. The patient was transferred to the recovery room and then the ward without any respiratory or hemodynamic complications. Histological examination of the aryepiglottic fold masses revealed they were granulation tissues. The patient was discharged from the hospital 3 days after the operation without complications.

## Discussion and conclusions

This case report presents our management of a patient with bilateral, mobile, and pedunculated masses on both aryepiglottic folds. From the collaborative preoperative evaluation and planning with the ENT surgeon, the main focus of airway management was to minimize the risk of damage, bleeding, or displacement of the masses while maintaining airway patency. These risks would have increased if the process of awake intubation, which includes topical anesthesia and flexible bronchoscopic intubation, did not proceed smoothly. Therefore, our airway management of this patient may seem to have strayed from the conventional gold standard for difficult airway management. Because the concept of a perceived difficult airway encompasses a wide range of circumstances, an individual approach may be more appropriate to ensure optimal decisions rather than a strict adherence to a uniform difficult airway algorithm (Apfelbaum et al. 2022).

First, awake intubation was not attempted, although difficult ventilation and intubation were predicted. Instead, tracheal intubation was attempted after induction of general anesthesia, including neuromuscular blockade. Although awake intubation has been cited as the gold standard for management of anticipated difficult airways, it may be prudent to choose between awake and post-induction airway management on a case-by-case basis (Apfelbaum et al. 2022). In addition, the literature is insufficient to evaluate the benefits or risks of maintenance versus ablation of spontaneous ventilation and the use of neuromuscular blockade to improve mask ventilation (Apfelbaum et al. 2022). Our assessment was that manual mask ventilation would be maintained after ablation of spontaneous ventilation and administration of neuromuscular blockade for this patient, as there were no signs of stridor or dyspnea in any position including supine position. Instead, concerns were raised about the risk of the patient’s hanging masses being dislodged due to upper airway reflexes, such as coughing and laryngospasm. Despite the relatively low likelihood of such an event occurring due to the patient’s own upper airway reflexes, we could not completely rule out the risk. This was particularly due to the fragility of the masses’ stalks, as confirmed by the experienced ENT surgeon’s preoperative examination. This could occur during the application of topical anesthesia for awake intubation or the process of endotracheal intubation using a flexible bronchoscope. It could even occur during post-induction intubation if the depth of general anesthesia was insufficient to suppress upper airway reflexes. To address this, adequate doses of propofol, remifentanil, and rocuronium were sequentially administered while ensuring that mask ventilation was maintained with ease. When the attending anesthesiologist determined that the patient was sufficiently anesthetized to suppress upper airway reflexes, a gentle videolaryngoscopy was performed to evaluate the feasibility of intubation.

To ensure the safety of airway management, the induction of general anesthesia was performed in preparation for a difficult mask ventilation and tracheal intubation. Our primary backup plan, in case of ventilation and intubation failure, was to awaken the patient. This strategy included preoxygenating the patient for over 5 min and preparing 16 mg/kg of sugammadex for the rapid reversal of rocuronium (Gambee et al. 1987, Sørensen et al. 2012). Our approach also included the administration of a single dose of propofol and a continuous infusion of remifentanil, each characterized by short half-life and short context-sensitive half-time, respectively. Therefore, we anticipated that spontaneous ventilation would resume well before the onset of hypoxemia. Additionally, in the event of patient emergence and oxygenation failure, an ENT surgeon was on standby to perform an emergency tracheostomy.

Second, intubation was performed with videolaryngoscopy instead of flexible bronchoscopy. Precise manipulation of the flexible tip of the bronchoscope is difficult, especially in the presence of supraglottic masses (Choi et al. 2010, Liew et al. 2015). In this case, manipulating a thin flexible bronchoscope to pass between the masses, push them aside, and advance into the glottic opening could require multiple attempts, increasing the risks of bleeding, damage, or displacement of the masses. In addition, during intubation using a flexible bronchoscope, the tip of the ETT is not visible. Therefore, it would be unclear whether the tumors were being displaced beyond the vocal cords due to the tube insertion. In this case, due to the narrow space between the bilateral masses and the glottal opening, advancing the ETT over the flexible bronchoscope could push the masses into the glottis. This poses a risk for serious complications, such as bronchial obstruction and collapse. Therefore, we chose to use a videolaryngoscope instead of a flexible bronchoscope for tracheal intubation. The videolaryngoscope offers better control because the curved blade tip can be manipulated more easily in the desired direction and placed more accurately within the intended anatomical structure (Choi et al. 2010). We carefully placed the tip of videolaryngoscope blade in the vallecula, which was free of pathology. This method allowed for full visualization of the ETT tip on the monitor. Furthermore, the styletted ETT afforded better directional control compared to the flexible bronchoscope. As a result, the ETT tip was precisely advanced under video control through the narrow space between the bilateral masses and into the glottic opening, leading to safe and successful intubation in just one attempt.

In conclusion, tracheal intubation using a videolaryngoscope after induction of general anesthesia, including neuromuscular blockade, may be feasible in patients with supraglottic masses who do not exhibit stridor or dyspnea. However, the decision must be based on a comprehensive preoperative evaluation and adequate preparation. It is also essential to have prearranged strategies in place to address potential challenges related to inadequate oxygenation and unsuccessful tracheal intubation. Integral to these preparations is the collaboration of a multidisciplinary team, which encompasses every stage from evaluation and planning to the execution of the procedure, thereby ensuring safe and effective airway management.

## Data Availability

The data and materials associated with the current study are available from the corresponding author on reasonable request.

## Abbreviations

ENT: Ear nose and throat
ETT: Endotracheal tube
